# Anti-GAD65 autoantibody levels measured by ELISA and alternative types of immunoassays in relation to neuropsychiatric diseases versus diabetes mellitus type 1

**DOI:** 10.3389/fneur.2023.1111063

**Published:** 2023-05-25

**Authors:** Shenghua Zong, Anita M. Vinke, Peng Du, Carolin Hoffmann, Marina Mané-Damas, Peter C. Molenaar, Jan G. M. C. Damoiseaux, Mario Losen, Rob P. W. Rouhl, Pilar Martinez-Martinez

**Affiliations:** ^1^Department of Psychiatry and Neuropsychology, School for Mental Health and Neuroscience (MHeNS), Maastricht University, Maastricht, Netherlands; ^2^Department of Neurology, Maastricht University Medical Center (MUMC +), Maastricht, Netherlands; ^3^Algarve Biomedical Center, Algarve Biomedical Center Research Institute, Faro, Portugal; ^4^Central Diagnostic Laboratory, MUMC+, Maastricht, Netherlands; ^5^Academic Centre for Epileptology Kempenhaeghe/MUMC+, Maastricht, Netherlands

**Keywords:** GAD65 autoantibodies, autoimmune encephalitis, epilepsy, diabetes type 1, neuronal autoantibodies, ELISA, immunohistochemistry, cell-based assay

## Abstract

**Background:**

Anti-GAD65 autoantibodies (GAD65-Abs) may occur in patients with epilepsy and other neurological disorders, but the clinical significance is not clear-cut. Whereas high levels of GAD65-Abs are considered pathogenic in neuropsychiatric disorders, low or moderate levels are only considered as mere bystanders in, e.g., diabetes mellitus type 1 (DM1). The value of cell-based assays (CBA) and immunohistochemistry (IHC) for GAD65-Abs detection has not been clearly evaluated in this context.

**Objective:**

To re-evaluate the assumption that high levels of GAD65-Abs are related to neuropsychiatric disorders and lower levels only to DM1 and to compare ELISA results with CBA and IHC to determine the additional value of these tests.

**Methods:**

111 sera previously assessed for GAD65-Abs by ELISA in routine clinical practice were studied. Clinical indications for testing were, e.g., suspected autoimmune encephalitis or epilepsy (neuropsychiatric cohort; *n* = 71, 7 cases were initially tested positive for GAD65-Abs by ELISA), and DM1 or latent autoimmune diabetes in adults (DM1/LADA cohort (*n* = 40, all were initially tested positive)). Sera were re-tested for GAD65-Abs by ELISA, CBA, and IHC. Also, we examined the possible presence of GAD67-Abs by CBA and of other neuronal autoantibodies by IHC. Samples that showed IHC patterns different from GAD65 were further tested by selected CBAs.

**Results:**

ELISA retested GAD65-Abs level in patients with neuropsychiatric diseases was higher than in patients with DM1/LADA (only retested positive samples were compared; 6 vs. 38; median 47,092 U/mL vs. 581 U/mL; *p* = 0.02). GAD-Abs showed positive both by CBA and IHC only if antibody levels were above 10,000 U/mL, without a difference in prevalence between the studied cohorts. We found other neuronal antibodies in one patient with epilepsy (mGluR1-Abs, GAD-Abs negative), and in a patient with encephalitis, and two patients with LADA.

**Conclusion:**

GAD65-Abs levels are significantly higher in patients with neuropsychiatric disease than in patients with DM1/LADA, however, positivity in CBA and IHC only correlates with high levels of GAD65-Abs, and not with the underlying diseases.

## Introduction

Glutamate decarboxylase 65 (GAD65) is an intracellular enzyme responsible for the synthesis of the inhibitory neurotransmitter γ-aminobutyric acid (GABA), which is present in neurons and also in the beta-cells in the pancreas (1, 2). As such, autoantibodies against GAD65 (GAD65-Abs) have been associated with not only several neuropsychiatric conditions, including stiff-person syndrome (SPS), epilepsy, limbic encephalitis, cerebellar ataxia, and paraneoplastic neurological syndromes (3), but also with type-1 diabetes mellitus (DM1) and latent autoimmune diabetes in adults (LADA), in which the presence of GAD65-Abs suggests autoimmune-induced destruction of the insulin-producing beta-cells in the pancreas.

GAD65-Abs levels are usually found to be much higher in the mentioned neurological disorders than in DM1 (1, 4, 5). Recently, a study suggested that GAD65-Abs levels higher than 10,000 U/mL could be used as a cut-off for immunotherapy in neurological disorders (6). Such levels are derived from the comparison among enzyme-linked immunosorbent assay (ELISA), cell-based assay (CBA), and rat brain-based immunohistochemistry (IHC) with sera from patients who initially tested positive for GAD65-Abs by ELISA, while ELISA negatives were not included (7). Considering the fact that CBA, or IHC, is able to identify autoantibodies that bind exclusively to antigens that are expressed in their natural conformation in their resident membranes, ELISA negative cases, in theory, have the chance of being positive by CBA or IHC.

It should be borne in mind, however, because of its intracellular location, that GAD65 as a pathogenic autoimmune target might be doubtful; GAD65-Abs could in fact be harmless bystander immunoglobulins, while other, genuinely pathogenic, but unknown, autoantibodies could be present in the same individual, causing an autoimmune disease (8, 9). These yet unidentified autoantibodies can be detected based on the sera staining on rat brain-based immunohistochemistry (IHC) (9). Besides, it has been reported that some cases had autoantibodies against GAD67, another isoform of GAD, even in the absence of GAD65-Ab (10–12).

In this study, we compared the sera reactivity to GAD65-Abs by CBA, IHC, and ELISA in a previously described neuropsychiatric cohort including ELISA positive and negative cases (13), with an additional diabetes cohort as disease control. We aimed to re-evaluate the background of the assumption that high GAD65-Abs levels are related to neurological disorders and lower levels are related to diabetes. To exclude other immunoreactivity and to evaluate the value of these tests in this clinical situation, we also tested for autoantibodies against conformational epitopes on GAD65, or solely against GAD67 by CBA, and neuronal autoantibodies that were not routinely tested in the clinic after positive findings in IHC.

## Methods

### Cohort

**Figure 1:**
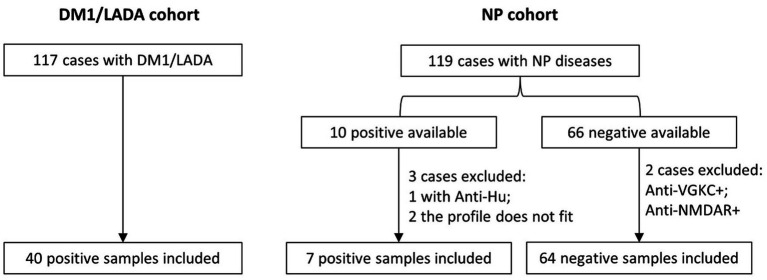
Procedure of the selection of blood samples and the basic subgroups for 3 different aims: (1) Comparison of the GAD-65 levels between positive samples of DM1/LADA and NP by ELISA (re-evaluation); (2) Comparison of the GAD-65-Abs detection methods between ELISA, CBA and IHC using both positive and negative cohorts. (3) Detection of GAD67-Abs by CBA and of other neuronal autoantibodies by CBA and IHC in all the mentioned groups.

We retrospectively included all patients from whom a GAD65-Abs test was ordered at Maastricht University Medical Centre (MUMC+) and Kempenhaeghe Epilepsy Centre between 2010 and 2014 (as reported previously) (13). In the initial study, in total, the cohort consisted of 117 patients with DM1/LADA and 119 patients with (possible) neuropsychiatric disorders (mostly epilepsy and/or encephalitis) who were tested for GAD antibodies during this period (13). For the current retrospective study, samples of the patients were selected in a process as shown in Figure 1. In brief, 40 GAD65-Abs positive sera from DM1/LADA patients were retrieved when available. Seventy six samples from patients with suspected autoimmune encephalitis/epilepsy were retrieved including 10 positives for GAD65-Abs, and 66 negatives for GAD65-Abs. We also took the results of previous tests for other anti-neuronal antibodies (from routine clinical practice) into account. We excluded three of these patients with positive results in these tests because they had the typical clinical presentation associated with the detected autoantibodies (one with anti-Hu and GAD65-Abs, and 2 cases negative for GAD65 but with anti-VGKC, and anti-NMDAR antibodies respectively). One patient with anti-VGKC and GAD65-Abs was not excluded, because the clinical syndrome was considered linked to the GAD65-Abs (and not to the anti-VGKC). In 2 patients the GAD65-Abs were not deemed to be related to the neuropsychiatric disease based on the initial and the follow-up evaluations (reassessed by AMV and RPWR), which were also excluded. This led to a final inclusion of 7 cases with GAD65-Abs and 64 cases negative for GAD65-Abs in the neuropsychiatric cohort (NP cohort) (Figure 1).

We retrieved the clinical symptoms at presentation, comorbidities, and previous history from the electronic patient files [as previously described in (13)]. The data are shown in Table 1. Ethical approval was obtained from the medical ethical committees of the two participating centers, MUMC+ and Kempenhaeghe (METC 15-4-002).

**Table 1:** Demographic and clinical characteristics of patients.

Main parameters	DM1/LADA	Neuropsychiatric cohort		*p*-value
Previous GAD65-Abs ELISA result	40 positive	9 positive	64 negative	
Age (mean/range)	38/4–68	33/15–64	40/1–83	NS
**Sex**				
Female	18 (55%)	5 (55.6%)	32 (50%)	NS
Male	22 (45%)	4 (44.4%)	32 (50%)	NS
children (<18 years)	9 (22.5%)	1 (11.1%)	8 (12.5%)	NS
Indication for GAD65-Abs test request of the neuropsychiatric patients*				
Refractory epilepsy		6 (66.7%)	28 (43.8%)	NS
Other neuropsychiatric disorders (encephalitis, movement disorders, psychotic disorders)		3 (33.3%)	36 (56.2%)	NS
Comorbid with DM1/LADA		6 (66.7%)	1 (1.6%)	0.0001
Tumor		0	4 (6.3%)	NS
Clinically diagnosed as GAD-Abs related neuropsychiatric disease		7 (77.8%)		
Treated with immunotherapy		4		
Response to immunotherapy		4		

GAD65-Ab, glutamate decarboxylase 65 autoantibodies; DM1/LADA, diabetes mellitus type 1 or latent autoimmune diabetes in adults; NS, no significant difference.

### Autoantibody detection methods

#### ELISA

As mentioned in our previous study, during routine clinical diagnosis, an ELISA for GAD65-Abs detection was performed at different national reference laboratories (all accredited according to national standards) using commercial ELISA kits following manufacturers’ instructions (13). Due to possible inter-laboratory differences, it is impossible to compare the levels of the used tests. To be able to compare the levels of the GAD65-Abs, we retested all samples using the commercial ELISA kit (RSR Limited, Cardiff, UK) in our laboratory following the same guidelines as above. Samples with levels above 2000 U/mL were further diluted 1 in 100 with phosphate-buffered saline (PBS) for a second test, and if still out of range further diluted 1 in 100 in PBS (1/10,000). Levels were expressed in units/mL (U/mL), levels below 5 U/mL were considered negative.

#### Cell-based assay for GAD65/67-Abs

Antigen-specific screening for autoantibodies against GAD65 and GAD67 was performed, respectively, using CBA as described in our previous study (14, 15). Basically, HEK293 cells were plated on coverslips coated with poly-D-lysine (#P7280, Sigma, St. Louis, United States) in 60 mm-culture plates (#628160, Greiner Bio-One, Alphen aan den Rijn, NL) in Dulbecco’s Modified Eagle Medium with 10% fetal calf serum, 4 mM L-glutamine and 100 U/mL penicillin-streptomycin and incubated overnight to attach. Cells were transfected with polyethyleneimine (#23966, Polysciences Inc. Warrington, PA, United States) and 4 μg expression vectors encoding the according antigen (plasmids pCMV6-XL5 containing human GAD65 or GAD67, a kind gift of Dr. Francesc Graus) and expression allowed for 22–26 h. Cells were fixed in 3.5% formaldehyde (#87837.180, VWR, Amsterdam, NL) for 10 min and permeabilized with 0.3% Triton-X-100 for 10 min. After blocking with 1% bovine serum albumin (BSA) for 1 h, cells were incubated with 40 μL human sera (diluted 1:40 in 1% BSA) for 1 h at room temperature. After that, secondary antibodies goat-anti-human-IgG-Alexa488 (1:1,000, # A11013, Invitrogen, Waltham, United States) were used for probing the autoantibody staining. Lastly, cover glasses were mounted with7 μL DAPI mounting medium (#H-1200, vector laboratories, CA, United States) and the results were evaluated with a BX51 Olympus microscope by two observers of which one was blinded of the sample’s information. A serum sample with antibodies both positive to GAD65 and GAD67 from a refractory epilepsy patient was used as positive control. The positivity had been initially tested by CBA using transfected cells and co-stained with antibodies from immunized rabbits specifically targeting GAD65 or GAD67, respectively. The two antibodies were rabbit anti-human GAD65 (1:1,000, 7309LB) and GAD67 (1:1,000, 10266/20B), a kind gift from Christiane Hampe (University of Washington). Additionally, a serum sample from a healthy individual was used as negative control (Supplementary Figure S1). Results were graded as strong positive, positive, weak positive and negative. All samples were tested once and positive samples were repeated at least once more for verification.

#### Immunohistochemistry (IHC) on rat brain

**Figure 2:**
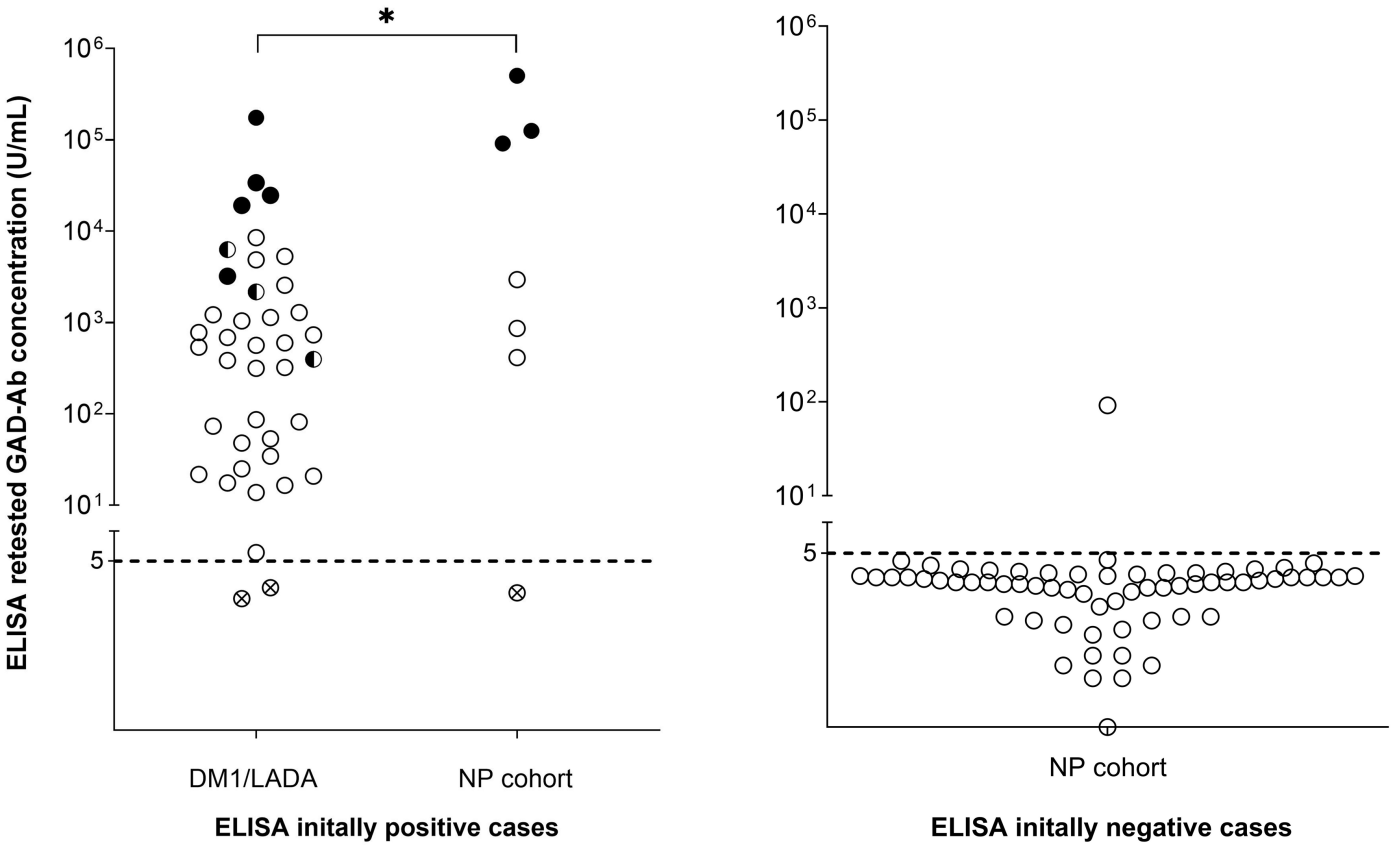
Analysis of GAD65-Ab by ELISA, CBA, and IHC in sera of patients with DM1/LADA and patients with neuropsychiatric disease. Every circle indicates an individual sample. Samples with GAD65-Abs levels above 5 U/mL were considered positive by ELISA. Half-black symbols indicate samples that were also positive by CBA (CBA+), and full-black symbols indicate cases positive by both CBA and IHC. Circles with cross symbols indicate samples that were retested negative in the ELISA initially tested positive cases. All samples with GAD65-Ab levels ≥10,000 U/mL were positive for both CBA and IHC, and patients with positive CBA and IHC were found in both DM1/LADA and NP groups. The difference in GAD65-Ab levels between patients with GAD-related neuropsychiatric disease and patients with DM1/LADA was significant (*p* = 0.02, Mann–Whitney *U* test).

Neuronal autoantibodies were identified by IHC on rat brain tissue following standard procedures as described in our previous studies (14, 15). Different from those reported by other groups that focus mainly on cerebellum staining pattern (16), for the GAD65-Abs detection by IHC, the hippocampus staining pattern was focused since the cerebellum molecular layer staining is not specific for GAD65, and not all the hillock area of the Purkinje cells would be revealed clearly in each section. In our hands, the hippocampus staining pattern gave specific results compared to the negative control as shown in our previous study (15). In brief, rat brains were fixed for 1 h in 4% paraformaldehyde and cryoprotected by incubating in 30% sucrose solution. Frozen brains were cut into 7-μm thick tissue sections using a Leica CM3050S cryostat and stored at −80°C. Sections were subsequently blocked with 0.3% H_2_O_2_ for 15 min followed by washing with PBS 3 times and blocking with 5% goat serum for 1 h at room temperature. Next, sections were incubated with 200 μL diluted human serum (1:200 in 5% goat serum) overnight at 4°C, followed by incubation with 200 μL biotinylated goat anti-human IgG (1:1,000, 109-066-008, Jackson laboratory) for 2 h at room temperature, followed by incubation with the same amount of ABC mixture (1:800 in TBS, # PK 6100 vector laboratories, CA, United States), for 1 h at room temperature and the reactivity developed using diaminobenzidine. A negative control from a healthy individual and a positive control serum for GAD65-Ab (serum from an autoimmune GAD65-Ab encephalitis patient, which was tested GAD65 and GAD67-Ab positive by CBA and gave a typical pattern on rat brain as compared to previous studies (15, 17)) and also our rabbit- anti-GAD65 antibody, see Figure 2. Images were taken by the VENTANA iScan HT slide scanner (20× objectives) and graded by 2 experienced observers separately (S. Zong, C. Hoffmann, or M. Damas) using the Ventana Image Viewer. Hippocampal reactivity of sera was ranked as negative (0), borderline (1), weak positive (2), strong positive (3), based on the intensity and contrast of the staining. Staining was repeated and only if repeatedly found positive with the same pattern by 2 observers, a final decision of positivity was made.

## Results

### GAD-Abs levels are higher in patients with neuropsychiatric disease than in patients with diabetes

To evaluate if the GAD65-Abs levels are substantially different between diabetes and GAD-related neurological disease, we compared results from the newly performed ELISA tests. We found that 38 out of 40 samples (95%) from the DM1/LADA cohort, and 6 out of 7 samples (85.7%) from the NP cohort were confirmed positive, whereas 63 out of 64 samples (98.4%) were confirmed negative in our hands (Figure 2; Table 2). In the neuropsychiatric group with confirmed positive ELISA results, 6 patients had a clinical diagnosis of GAD65-Abs related neuropsychiatric disease (epilepsy or encephalitis). The GAD65-Abs level in patients diagnosed with GAD-related neuropsychiatric disease (*n* = 6) was higher than in patients with DM1/LADA (*n* = 38), with statistical significance: median 47,092 U/mL vs. 581 U/mL; *p* = 0.02, Mann-Whitney *U* test (Figure 2).

**Table 2:** Comparison of ELISA, CBA, and IHC results.

	ELISA initially positive cases		ELISA initially negative cases	
	DM1/LADA (*n* = 40)	NP (*N* = 7)	NP (*n* = 64)	
ELISA retested GAD65-Abs+*	38 (95%)	6 (85.7%)	1 (1.6%)	
CBA GAD65-Abs+	8 (20%)	3 (42.9%)	0	
IHC GAD65-Abs+	5 (12.5%)	3 (42.9)	0	
IHC positive for other Abs	2 (5%)	0	2 (3.1%)	
	CBA GAD65-Abs+		CBA GAD65-Abs−**	
	DM1/LADA (*n* = 8)	NP (*N* = 3)	DM1/LADA (*n* = 32)	NP (*N* = 4)
CBA GAD67-Abs+***	6 (75%)	3 (100%)	0	0
IHC GAD65-Abs+	5 (62.5%)	3 (100%)	0	0

*GAD65-Abs+: Positive for GAD65 autoantibodies;

**GAD65-Abs−: Negative for GAD65 autoantibodies

***GAD67-Abs+: Positive for GAD67 autoantibodies.

### Only samples with high levels of GAD65-Abs by ELISA are positive by CBA and IHC

To assess a possible added value of CBA and IHC for the detection of GAD65-Abs, all samples were further tested by these 2 methods; the results were compared to the ELISA results between the two studied cohorts (Figures 2 3; Table 2). 42.9% (3 out of 7) of the ELISA positive samples from NP patients were positive for both CBA and IHC (GAD65 Abs levels between 91,238 U/mL and 503,001 U/mL), whereas 20% (8 out of 40) of ELISA positive samples from DM1/LADA patients had positive CBA(the lowest GAD65 Abs level was 396 U/mL), 5 of which were also positive by IHC (the lowest GAD65 Abs level was 3,219 U/mL), with no significant prevalence difference between the 2 cohorts. Notably, all samples (*n* = 7) with antibody levels above 10,000 U/mL were positive by CBA and IHC, regardless of cohorts difference (Figure 2). All (*n* = 64) ELISA negative samples were negative in IHC and CBA (Table 2).

**Figure 3:**
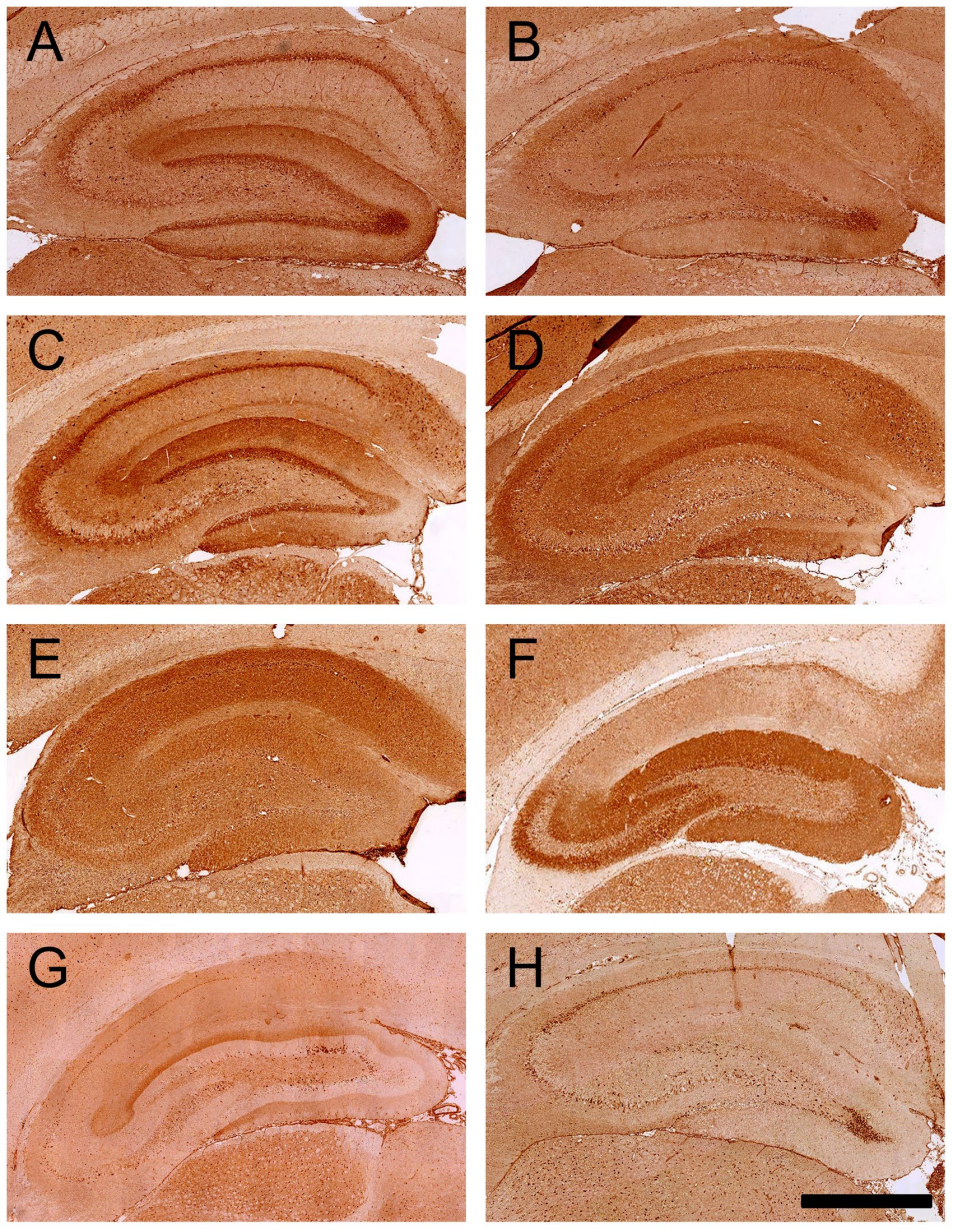
IHC staining patterns on rat brain hippocampus given by sera of patients with DM1/LADA and neuropsychiatric disease. Commercial antibodies to GAD65 or GAD67, or human sera were incubated on rat brain slices, followed by biotinylated secondary antibodies, ABC kit, and DAB to develop the color reaction. **(A)** Commercial antibodies specific to GAD65 showed intracellular granular staining in the hippocampus and neuropil staining in the outer layer of the DG region. **(B)** The commercial antibody directed against GAD67 did not give strong staining in the hippocampus region. **(C)** Positive serum with high GAD65-Abs levels by ELISA (>10,000 U/mL) gave the same pattern as the commercial GAD65-Abs. **(D–G)** Two samples from DM1/LADA patients (Case 1 and Case 2) and 2 samples with neuropsychiatric disease (Case 3 and Case 4) gave strong neuropil staining in the hippocampus (**G** represents staining performed at another time point; therefore, the background staining is different). **(H)** Negative control serum from a healthy individual showed overall background staining (scale bar = 500 μm).

### Besides GAD65-Abs, other neuronal autoantibodies are present in both patient groups

To test for the presence of other neuronal autoantibodies, all samples were tested for GAD67-Abs by CBA and other neuronal autoantibodies by IHC. Nine samples tested positive for GAD67-Abs; all were also positive for GAD65-Abs by CBA (six were from the DM1/LADA cohort and three from the NP cohort, Table 2).

Samples from four cases gave staining patterns different from typical GAD65-Abs by IHC (Table 2; Figures 3D–G). Two cases were patients with LADA (GAD65-Ab levels were 779 U/mL and 1,136 U/mL, respectively). One of these patients had an anxiety disorder, and by IHC there was mainly staining in the molecular layer of the dentate gyrus (DG) and the subiculum regions; the other patient did not have any neuropsychiatric symptoms and showed diffuse staining over the hippocampus (Figures 3D,E; Table 3 case 1 and case 2, respectively).

**Table 3:** Four Clinical characteristics of four cases with other neuronal autoantibodies identified by IHC.

	Sex	Age	ELISA GAD65-Ab	CBA GAD65/67-Ab	IHC	Diagnosis	Comorbidity	Treatment	Response to treatment
Case 1	M	50	779.1	Negative	Weak positive to unknown antigens	LADA	Anxiety	n/a	n/a
Case 2	M	65	1136.5	Negative	Weak positive to unknown antigens	LADA	No	n/a	n/a
Case 3	F	51	3.4	Negative	Anti-mGluR1	Refractory epilepsy (anti-mGluR1 encephalitis)*	No	Valproic acid (IVIG)*	Seizure reduction; (further reduction)*
Case 4	M	83	0.3	Negative	Weak positive to unknown antigens	Encephalitis	No	Fenytoin, Levetiracetam	Seizure reduction

GAD65/67-Abs, glutamate decarboxylase 65/67 autoantibodies; ELISA, enzyme-linked immunosorbent assay; CBA, cell-based assay; IHC, immunohistochemistry; Anti-Hu, anti-Hu autoantibodies; Anti-mGluR1, autoantibodies against metabotropic glutamate receptor 1; LADA, Latent Autoimmune Diabetes in Adults; n/a, not applicable.

*Anti-mGluR1 encephalitis was the final diagnosis of case 3 after the IHC results confirmed by CBA, thus the patient was treated further.

Also, in the GAD65-Abs negative patients with the neuropsychiatric disease, we could identify cases with reactivity in IHC. In case 3, there was strong reactivity in the CA3 and DG area of the hippocampus and the molecular layer of the cerebellum (different from the 2 patterns found in the LADA cases mentioned above, seen in Figure 3F; Table 3 case 3). This patient had epilepsy. The staining pattern was similar to anti-mGluR1 as previously reported (18) and we confirmed this by mGluR1 CBA. The details of this case can be found in our recently published case report (19). In case 4, the patient had encephalitis, and the sample showed a staining pattern similar to case 1 (Table 3).

## Discussion

We found that patients with neuropsychiatric disease tend to have higher levels of GAD65-Abs in their serum than DM1/LADA patients, though there is a substantial overlap. This suggests that the antibody levels may be a discriminative factor between the two clinical groups. However, neither the results of IHC nor CBA assays show a significant difference between the two groups.

Previously, studies showed that GAD65-Abs levels in patients with confirmed GAD65-Abs related neuropsychiatric diseases were higher than those in patients with DM1. Baekkeskov et al. in 1990 firstly described that the level of the GAD65-Abs in stiff person syndrome was 10–200 times higher than in DM1 patients on the basis of an immunoprecipitation method, and later Honnorat et al. in 2001 found similar results in patients with cerebellar ataxia compared to patients with DM1 using RIA and IHC (with the staining pattern of GAD65-Abs on rat cerebellum) (1, 20). In both studies, however, the confirmation of the GAD65-Abs was done with two methods in patients with neuropsychiatric disease, but only with one for patients with DM1.

GAD-65 analysis in neuropsychiatric cohorts, e.g., epilepsy or autoimmune encephalitis were tested but the quantification of the results contradicted each other (4, 5). Since there are no clear criteria to diagnose and define GAD-related neuropsychiatric disorders, the selection procedures in these studies were different, which may have contributed to the conflicting results. Within the inclusion cohort in the present study, our findings tend to support that the cut-off value of autoantibody levels (GAD65-Abs levels) for clinical significance in patients with neuropsychiatric disease cannot be as strict as claimed in earlier studies (7). The limitation of applying this cut-off is that patients from DM1/LADA with high GAD65-Abs titers, if combining with neurological or psychiatric disorders, might be suspicions, while this is not supported by the current study because only 2 of the 5 DM1/LADA patients with high titers GAD65 antibodies (positive for all the assays) had psychiatric disorders. Furthermore, considering the relatively high comorbidity of DM1 in patients with epilepsy (21), the clinical relevance of GAD65-Abs for the neuropsychiatric disease (mainly epilepsy in this case) in these patients remains doubtful.

Measurements of GAD65-Abs in cerebrospinal fluid (CSF) and serum, as well as analysis of intrathecal synthesis were proposed to be important to identify GAD associated autoimmunity (16), Based on the dynamic turnover rate of immunoglobulin G (IgG) between serum and CSF, around 1% of the serum levels of these antibodies would enter the CSF (18, 22). In case of disruption of the blood–brain barrier, the CSF antibody levels would be higher. According to this theory, diabetes patients with high antibody levels would also show positive results in CSF. On the other hand, intrathecal synthesized antibodies would leak to serum as well and lead to a relatively low but positive serum antibody levels. Thus, as long as measurements of GAD65-Abs in serum is the starting point in the clinical practice, a positive result should always lead to further investigation, while the level should not be used as an absolute marker for diagnosis. This is partially supported when comparing the clinical features and treatment response between the triple positive patients and the ELISA only patients, of which no obvious correlation with the titers (Supplementary file Supplementary Tables S1, S2).

Another point is the possibility that in the different related disorders the GAD65-Abs are directed against different antigenic regions of GAD65 (8, 23). However, until now literature has not provided a practical method to identify antibodies targeting different antigen binding sites for a clear-cut diagnosis of GAD65-related diseases. Here, we limit our discussion to the comparison of the current clinical methods: CBA, and in some laboratories also IHC, are used frequently in the detection of neuronal autoantibodies. We found positive results only by CBA and IHC when the concentrations of GAD65-Abs are high, and there was no additional value in the use of IHC or CBA to distinguish the 2 clinical groups. Apparently, the latter assays seem to be less sensitive for GAD65-Abs.

Whether other co-existing autoantibodies might be more relevant contributors to the disease than GAD65 should be assessed case by case. The clinical relevance of neuronal autoantibodies, like anti-NMDAr, anti-LGI1, and anti-GABAbr, is mostly clear, whereas neuronal autoantibodies with only weak reactivity to the brain (in IHC) might be common and only related to aging (14, 24). In our study, 2 cases with unknown IHC patterns had LADA and one of these also suffered from anxiety. In these cases, further studies analyzing a possible correlation between the unknown neuronal autoantibodies and the anxiety symptoms in LADA are needed, because also clinically, diabetes and anxiety disorders have high co-occurrence (25, 26).

Our study has some limitations: Firstly, the matched CSF samples were not available for testing. Secondly, the rate of false positive IHC results for detecting novel neuronal autoantibodies is unknown. Thus, the autoantibodies found positive only by IHC would always need further confirmation with more specific methods. Furthermore, we were dependent on the availability of sera in the archives of the participating centers, which led to a relatively small sample size of patients with neurological GAD65-Abs related diseases leading to a somewhat skewed comparison between the groups. Even though the rarity of this disease makes it difficult to have substantial number of patient samples, a larger sample size would increase the statistical power.

In conclusion, we reconfirmed a marked difference between GAD65-Abs levels between patients with neuropsychiatric disease and patients with DM1/LADA, whereas positive results in CBA and IHC overlap in both groups. Consequently, the clinical significance of the identification of GAD65-Abs in patients with neuropsychiatric disease remains unclear, even in patients with high levels of antibodies. We also recommend a strict correlation to the clinical symptoms and the demonstration of absence of other neuronal antibodies by practical methods such as CBA and/or IHC.

## Data availability statement

The raw data supporting the conclusions of this article will be made available by the authors, without undue reservation.

## Ethics statement

Ethical approval was obtained from the medical ethical committees of the two participating centers, MUMC+ and Kempenhaeghe (METC 15-4-002). Written informed consent from the participants’ legal guardian/next of kin was not required to participate in this study in accordance with the national legislation and the institutional requirements.

## Author contributions

SZ contributed to the study design, acquisition, analysis and interpretation of the data, and manuscript drafting and revision. AV contributed to the acquisition, analysis and interpretation of the data (especially to the clinical information collection and analysis), and revision. PD contributed to the analysis and interpretation of the data, and revision (specifically focusing on the additional experiments’ performance and results analysis during this stage). CH contributed to the study design, acquisition, analysis and interpretation of the data and the revision. MM-D contributed to the study design, acquisition, analysis and interpretation of the data. PM contributed to the interpretation of the data and the revision. JD contributed to the study design, acquisition and interpretation of the data (especially on the sample collection), and revision. ML contributed to the study design, interpretation of the data, and manuscript revision. RR contributed to the study design, analysis and interpretation of the data, and critical revision of the manuscript. PM-M contributed to the study design, acquisition, analysis, and interpretation of the data, manuscript and revision, and has access to the data in the study and takes responsibility for the integrity and data analysis. All the authors contributed to the critical revision of the manuscript for important intellectual content.

## Funding

The authors are thankful for the financial support received from the following funding organizations: PM-M was supported by an Aspasia/NWO grant (015.011.033), and SZ received CSC scholarship (201507720015) and Kootstra Talent Fellowship (Fall, 2019), which support his research in Maastricht University. CH and MM-D were supported by Kootstra Talent Fellowships.

## Conflict of interest

The authors declare that the research was conducted in the absence of any commercial or financial relationships that could be construed as a potential conflict of interest.

## Publisher’s note

All claims expressed in this article are solely those of the authors and do not necessarily represent those of their affiliated organizations, or those of the publisher, the editors and the reviewers. Any product that may be evaluated in this article, or claim that may be made by its manufacturer, is not guaranteed or endorsed by the publisher.
