# Supplementary material for: Anti-GAD65 autoantibody levels measured by ELISA and alternative types of immunoassays in relation to neuropsychiatric diseases versus diabetes mellitus type 1
Source: Front Neurol. 2023 May 25;14:1111063. doi: 10.3389/fneur.2023.1111063 (PMC10248002; doi:10.3389/fneur.2023.1111063)
Supplement: Supplementary file 1 [file Data_Sheet_1.PDF]

Supplementary: Titration for GAD65 autoantibodies (GAD65-Abs)

Methods:

After the qualification study by Cell-based assays (CBA), immunohistochemistry (IHC), and the quantification study by ELISA, 7 triple positive GAD65-Abs samples (tested positive by the three methods mentioned above) with enough volume left were further titrated by IHC and CBA.

1. Titration for GAD65-Abs by IHC
   The IHC titration of GAD65-Abs for each sample was done only once, with the dilution starting point 1 in 200, dilution factor 2, and ending at either 1:400 (for the weak typical pattern samples), 1: 800 (for the positive typical samples), or 1:1600 (for the strong positive samples), depending on the initial IHC score. The staining procedure was the same as described in the main content. A sample from a healthy individual was used, followed by the sample dilution series (1:200, 1:400, 1:800, and 1:1600). The results from the same sample were compared after scanning the images. And the endpoint of the dilution was discussed and decided by 2 observers when the staining pattern and intensity became weak/borderline positive.
2. Titration for GAD65-Abs by CBA:
   Basically, HEK293 cells were plated and transfected with GAD65, fixed, permeabilized, and blocked as mentioned in the main content of the method part, cells were incubated with 40 µL of diluted human serum for 1 hour at room temperature (for each sample, 6 dilutions were initially used, with dilution factor 2, starting dilution was 1 in 50). After that, secondary staining and mounting steps were followed, the results were checked as described in the method part of the main manuscript. Additionally, a serum sample from a healthy individual, with the same dilution series was used as a negative control. All samples were tested once. After the first round of titration, the remaining positive samples at the dilution of 1 in 1600 were further diluted for another 3 times, with dilution factor 2, starting dilution was 1 in 3200. Again, samples that remained positive of the second round titration were further tested with dilution factor 2, starting dilution was 1 in 20,000, till 1 in 160,000. The titration endpoint is when the staining becomes weak/borderline positive.

Results:

The titration results of those 7 samples were shown in the supplementary Table 1:

Supplementary Table 1: CBA and IHC titration for the triple-positive samples

Samples	Cohorts	ELISA titer	CBA titration	IHC titration
Sample 1	DM1/LADA	24659,5	1: 50	>1: 400
Sample 2	DM1/LADA	33904,5	1: 800	1: 400
Sample 3	DM1/LADA	174965,0	1: 6400	1: 1600
Sample 4	DM1/LADA	19207,0	1: 50	1: 200
Sample 5	NP	125391,6	1: 12800	>1: 1600
Sample 6	NP	91238,2	1: 20000	>1: 1600
Sample 7	NP	503001,3	1: 160000	>1: 1600

Representative results of the IHC and CBA titration were shown in Supplementary Figure 1:

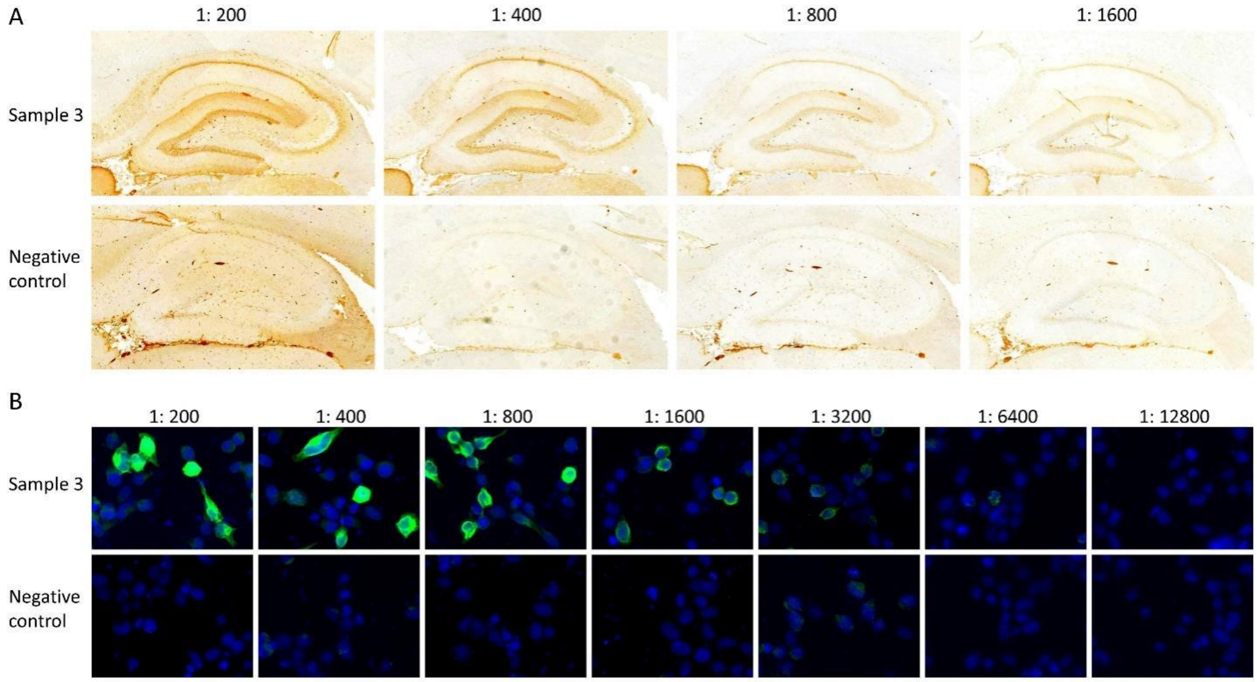

Supplementary Figure 1: The titration of sample 3 by immunohistochemistry (IHC) and Cell-based assay (CBA). Panel A: Titration of sample 3 by IHC with 4 different dilutions (1:200, 1:400, 1:800, and 1:1600). Both the staining pattern of GAD65 and the background faded away as increasing of the dilution. The staining intensity of sample 3 at 1: 1600 was weak/borderline positive, and thus was defined as the endpoint of the dilution. Panel B: Titration of sample 3 by CBA with 7 different dilutions (from 1: 200 till 1: 128000, with a dilution factor 2). The staining of GAD65 on transfected cells became as weak/borderline positive at dilution 6400 and thus was defined as the endpoint of the dilution.

Discussions:

Basically, the higher the ELISA titer was, the sample remained still positive after a higher dilution. While samples with similar ELISA titers (Sample 1 and Sample 2; Sample 5 and Sample 6), the titration of CBA or IHC showed contradicting results from the ELISA titers. Notice that this trend does not have a linear correlation, which might be contributed by that the calculation for ELISA titer is totally different from the subjective observation of CBA and IHC results under the microscope.

Supplementary Table 2: Clinical features of patients that were diagnosed with GAD-Abs-related neurological diseases (ELISA, CBA, and IHC triple-positive cases versus ELISA-positive only cases)

Groups	Patients*	age	sex	Duration of illness	with DM1 or not	ELISA titers	main symptoms	Immunotherapy	Treatment response
Triple-positive patients	Patient 5	27	male	13 years	Yes	125391,6	seizures	Immunoglobulins, azathioprine, mycophenolate	moderate
	Patient 6	25	female	5 years	No	91238,2	seizures	no immunotherapy	not applicable
	Patient 7	64	female	>30 years	Yes	503001,3	seizures	no immunotherapy	not applicable
ELISA-positive only patients	Patient 9	18	male	3 years	Yes	861,8	partial seizures, memory complaints	plasmapheresis, mycophenolate, immunoglobulins	moderate
	Patient 10	15	male	1 year	Yes	423,6	seizures, cognitive problems (learning problems)	immunoglobulins, prednisolone	good
	Patient 11	29	female	7 years	Yes	2946	Partial seizures	plasmapheresis, immunoglobulins	good

*The patient's code is with the sample's code in supplementary table 1.
